# Barriers Associated with Seasonal Influenza Vaccination among College Students

**DOI:** 10.1155/2016/4248071

**Published:** 2016-03-24

**Authors:** Stephanie M. Benjamin, Kaitlin O. Bahr

**Affiliations:** Department of Health Sciences, California State University, Northridge, 18111 Nordhoff Street, Northridge, CA 91330-8285, USA

## Abstract

Influenza can spread rapidly on college campuses because of high-density living conditions and frequent social interactions. However, seasonal influenza vaccination rates on college campuses are low. The purpose of this study is to identify barriers associated with receipt of the seasonal influenza vaccination. Questionnaires were completed by a convenience sample of 383 undergraduate students in January 2014. Data were analyzed to identify barriers associated with receiving the seasonal influenza vaccine. Only 20.6% of students reported receiving the vaccine within the last 6 months. Among students who did not receive the vaccine, 47.8% believed they would get influenza from the vaccine, 41.6% believed the vaccination may have dangerous side effects, and 39.6% believed they were not at risk for contracting influenza. The majority of nonvaccinated students did not believe cost of the vaccine or access to the vaccine were barriers. Many college students are not receiving the seasonal influenza vaccine, representing an important area for improvement. Understanding potential barriers associated with receipt of this vaccine is important for identifying and creating effective public health education programs and campaigns. There is a need for enhanced vaccination education efforts among college students, particularly with respect to the safety and importance of this vaccine.

## 1. Introduction

Influenza virus can be a serious and potentially deadly disease in persons of all ages. Outbreaks of influenza on college campuses are common because influenza has the ability to spread easily within student populations. Investigations of outbreaks among subgroups of college students have shown attack rates as high as 73% [1–4]. It has also been documented that students who live on campus are at greater risk of contracting influenza [5]. Although the influenza-attributable hospitalization rate for college students is low, the potential burden of a large outpatient population is substantial. Illness due to influenza can adversely affect students' academic performance and class attendance and increase antibiotic use and health care services [4, 6, 7]. To address this burden, the Centers for Disease Control and Prevention's Advisory Council on Immunization Practices currently recommends annual influenza vaccination for all persons aged six months and older, which includes all college students [8].

The seasonal influenza vaccine has been shown in randomized trials to be highly effective in preventing influenza among healthy college students [9]. However, recent studies have shown seasonal influenza vaccination rates among college students are low, with receipt of the vaccine ranging within 12–30% [10–12]. These studies have also identified predictors of receipt of the vaccine in college students, which include being a lower-classman, attending a private school, having a parent who graduated from college, participating in an academic club or honor society, participating in community service or volunteer work, and using e-mail often [10, 11]. Other predictors include not being employed and students who were considered to be at higher risk for medical complications due to influenza [12]. A few studies have examined attitudes and beliefs towards the seasonal influenza among students. A study conducted among undergraduate students in 2007 found that 29% of students believed they would get influenza as a result of receiving the vaccine [10]. Additionally, a study conducted among medical students in England found the strongest barriers to acceptance of the vaccine included fear of side effects, lack of vaccine information, lack of perceived risk, and inconvenience [13]. The purpose of this study is to identify factors and barriers associated with the receipt of the seasonal influenza vaccine among undergraduate students at a public university.

## 2. Methods

Utilizing a cross-sectional design, a convenience sample of students were recruited from a common student gathering location on the campus of California State University, Northridge (CSUN), during a one-week period in January 2014. CSUN is a diverse university community of 38,310 students located in Los Angeles' San Fernando Valley. Of the 33,771 undergraduates, 55% are female, 40% are Hispanic, the mean age is 23, and 49% are low income students [14]. Respondents met the study inclusion criteria if they were undergraduate students, at least 18 years of age and able to both read and write in English. The study was approved by the Institutional Review Board at CSUN on November 19, 2013.

Participants were asked to complete a questionnaire including questions on basic demographic information (age, sex, race/ethnicity, on/off campus living situation, and year of study) and health care related information including whether or not they currently had health insurance, when they had last visited a medical provider, and if they had received any encouragement or information about the seasonal influenza vaccine from a health professional (including a physician, nurse, or the campus student health center). The questionnaire also inquired about previous and current seasonal influenza vaccination history (ever received, received within the past 6 months, and planned to receive this season). Respondents who did not report being vaccinated during the current influenza season were asked about their attitudes regarding seasonal influenza vaccination, including beliefs about cost, access, and importance and risks of vaccination. Attitudes were assessed using respondents' level of agreement with statements about the seasonal influenza vaccine, using a Likert scale from 1 = strongly disagree to 4 = strongly agree. Questions on attitudes about the vaccine were adopted from a previous study conducted on a college campus to examine factors and barriers associated with receipt of the influenza vaccine [10]. As per the study inclusion criteria, responses from 383 participants who identified themselves as undergraduate students were used for analysis. Undergraduate students were chosen as the target population in the study design to provide comparability with other studies focused on college students. Analyses were completed using SAS software (SAS, Inc., V.9.4). Descriptive statistics were calculated for the demographic variables of interest. Differences in reported vaccination coverage between demographic factor categories were calculated using *t*-tests (age) and *χ* ^2^ tests (race/ethnicity, campus residence, and year of undergraduate study), with a significance level of 0.05. A multivariate logistic regression analysis was conducted to examine the association between the three health care related factors included in the study questionnaire and receipt of the seasonal influenza vaccine. The health care related factors included in the multivariate model, which were independently associated with receipt of the vaccine, were whether or not the participants currently had health insurance, when they had last visited a medical provider, and if they had received any encouragement or information about the vaccine from a health professional. Response means and standard deviations as well as percentage of agreement (either “Agree” or “Strongly Agree”) with statements regarding the seasonal influenza vaccine were calculated and attitudes/barriers to vaccination were ranked by order of percentage agreement.

## 3. Results

There were a total of 317 undergraduate students at CSUN who completed the questionnaire and included information on whether or not they had received the influenza vaccine in the last 6 months. Of this group, the mean age was 21 years, 55.8% were female, 45.4% were Hispanic, 82.6% reported residing off campus, 37.2% were in their first year, 50.8% reported seeing a medical provider within the last six months, 59.6% reported being encouraged to receive the seasonal influenza vaccine, and 72.2% reported having health insurance.

Descriptive statistics for demographic variables, stratified by reported receipt of the seasonal influenza vaccine, are presented in Table 1. Approximately 20% of respondents reported receiving the seasonal influenza vaccine in the last 6 months. The only demographic factor that was significantly associated with receiving the seasonal influenza vaccine included year of undergraduate study (*p* < 0.02).

Multivariate analysis of health care related factors identified as having an association with reported receipt of the seasonal influenza vaccine is presented in Table 2. Each of the health care related variables we examined had a statistically significant association with receipt of the vaccine, with the strongest association observed relating to current health insurance coverage (OR: 5.62, 95% CI [1.92–16.46]).

The percent agreement of responses to statements regarding the seasonal influenza vaccine among students who did not receive the vaccine is presented in Table 3. Issues related to safety had the highest percent agreement, followed by issues related to perceived importance. Among students who did not report being vaccinated, 47.8% of students agreed with the statement “I believe that as a result of the flu shot I may actually get the flu,” 41.6% agreed with the statement “I believe that vaccines may have dangerous side effects,” and 39.6% agreed with the statement “I do not believe I am in danger of contracting the flu.” Issues related to cost and access to the vaccine had the lowest percent agreement. Only 22.4% of students agreed with the statement “Vaccines are too expensive for me right now” and 19.4% of students agreed with the statement “I do not know where to receive a flu vaccination.”

## 4. Discussion

The results of this study show a low (20.6%) reported receipt of the seasonal influenza vaccine among undergraduate students at a large, diverse public university in Southern California. These findings are consistent with previous studies examining reported seasonal influenza vaccination rates among college students [10–12]. Also consistent with other studies were demographic and health care related characteristics associated with receipt of the vaccine, as well as barriers to receipt of the vaccine.

When identifying characteristics associated with receipt of the seasonal influenza vaccine, gender and race/ethnicity were not found to be statistically significant, which is consistent with previous studies [10]. Year of undergraduate study, however, was found to have a statistically significant relationship with receipt of the seasonal influenza vaccine (*p* < 0.02). The data showed that freshmen and sophomores were more likely to receive the vaccine, which is consistent with the literature [10, 11]. This may be a result of younger students being more likely to visit a physician, having health insurance, or being more likely to be encouraged to receive the vaccine. This finding indicates that interventions targeted to upper classmen might have a significant impact on vaccination rates.

The strong association between reported receipt of the vaccine and health insurance coverage indicates an important factor that, when modified, may have an impact on improving seasonal influenza vaccination coverage. Possessing health insurance has been shown to be associated with higher levels of influenza vaccine coverage in the general population [15]. Health insurance coverage might also affect how likely an individual is to visit and be encouraged to receive the seasonal influenza vaccine from a medical provider, both factors also found to be strongly associated with receipt of the vaccine [15, 16]. While college-aged students traditionally have had one of lowest reported health insurance coverage rates, hopefully the Affordable Care Act components, extending parental health insurance coverage for dependents to age 26 and making coverage more affordable for lower income individuals, will help to increase coverage among undergraduate college students. This could have a significant impact on increasing vaccinations in this important population.

A high proportion of students reported beliefs that the vaccine may have dangerous side effects and they might actually get influenza as a result of the flu shot. These safety concerns about the seasonal influenza vaccine represented the most frequently reported barriers to receipt of the vaccine. The Centers for Disease Control and Prevention (CDC) produces Vaccine Information Statements (VIS) required by law to be distributed with each seasonal influenza vaccination given, detailing benefits and risk of the vaccine [17]. The influenza VIS clearly states that an individual is not at risk of contracting influenza from the injected inactivated influenza vaccine and that there is a very remote chance of serious side effects in most populations [17]. While this may be providing critical information for those choosing to receive the vaccine, findings from this study demonstrate that people not choosing to receive the vaccine have substantial misconceptions about the risk of vaccinations that need to be addressed. It may be necessary to provide additional information, aside from the VIS, about the vaccine that is more targeted to college students to ensure they are able to make informed decisions regarding vaccination, which may lead to increases in vaccination rates in this population.

Many participants also agreed with statements indicating they did not believe they were at risk of contracting influenza and they were not informed that flu vaccines might be important. This represents an important gap in information that can be addressed. As mentioned earlier, previous studies have found seasonal influenza attack rates on college campuses as high as 73% [1–4]. College students do represent a vulnerable population to seasonal influenza because of their close living conditions in dormitories and high levels of social contact with their peers, which reinforces the critical need to educate college students about the importance of vaccination [11, 18, 19].

A minority of participants who did not receive the seasonal influenza vaccine felt that the cost of the vaccine was prohibitive and that they did not know where to receive a vaccine. This indicates that the cost of and access to seasonal influenza vaccination might not be the most important barriers to receipt of the vaccine in this population. It is important that vaccination costs remain practical for college students and campaigns educating this population regarding locations for vaccine receipt remain effective. However, future efforts to increase vaccination rates in this population may be most effective if focused on vaccine safety and importance.

An objective of Healthy People 2020 is to increase the percentage of adults aged 18 years and older who are vaccinated annually against seasonal influenza to 70% [20]. The vaccination rate in this college population would need to more than triple to meet the Healthy People 2020 target. These findings strongly emphasize the need for public health interventions that are effective in promoting and educating about the seasonal influenza vaccine among college students.

A limitation of this study was the use of a convenience sample. It could be that students who had more of an interest in the seasonal influenza vaccine were more inclined to participate in the study. The mean age of this sample was less than the mean age of the undergraduate population (21 versus 23 years of age) and this study as well as others show that underclass students are more likely to be vaccinated [10, 11]. Therefore, we may be overestimating the actual seasonal vaccination rate.

## 5. Conclusions

Findings from this study support the need for public health interventions to educate college students about the seasonal influenza vaccine, particularly with respect to the safety and relevance of the vaccine among this population. Increased efforts to educate college students about the risks and importance of the vaccine may serve to minimize widely held misconceptions about the vaccine. Successful education efforts may lead to important reductions in morbidity and mortality related to influenza among students and their families.

## Figures and Tables

**Table 1:** Demographic and health related characteristics of respondents by receipt of seasonal influenza vaccine (within the past 6 months).

	Received the vaccine *n* (%)	Did not receive the vaccine *n* (%)	*p* value^*∗*^
Total	79 (20.6)	238 (62.1)	—
Age (mean)	21.1	21.1	0.979
Sex			
Male	40 (50.6)	99 (41.6)	0.169
Female	39 (49.4)	138 (58.0)	
Race/ethnicity			
White/Caucasian	11 (13.9)	49 (20.6)	0.719
Black/African American	8 (10.1)	25 (10.5)	
Hispanic/Latino	40 (50.6)	104 (43.7)	
Asian/Pacific Islander	13 (16.5)	40 (16.8)	
Other	6 (7.6)	16 (6.7)	
*Missing/declined to State*	1 (1.3)	4 (1.7)	
Campus residence			
On campus	12 (15.2)	37 (15.6)	0.962
Off campus	65 (82.3)	197 (82.8)	
Year of undergraduate study			
Freshman	35 (44.3)	83 (34.9)	0.017
Sophomore	15 (19.0)	23 (9.7)	
Junior	13 (16.5)	69 (29.0)	
Senior	16 (20.3)	63 (26.5)	

^*∗*^ *p* values were calculated using *t*-tests (age) and *χ* ^2^ tests (race/ethnicity, campus residence, and year of undergraduate study), with a significance level of 0.05.

**Table 2:** Multivariate analysis of factors associated with reported receipt of the seasonal influenza vaccination (within 6 months preceding data collection).

	Odds ratio [95% confidence interval]
Saw a medical provider within the last 6 months	1.91 [1.02–3.57]
Encouraged by a health professional to receive the vaccine	4.21 [1.99–8.88]
Have health insurance	5.62 [1.92–16.46]

**Table 3:** Potential barriers to receipt of the seasonal influenza vaccine among students who reported not receiving the vaccine within the 6 months preceding data collection.^*∗*^

	*n*	Mean (sd)	% agreement	Rank^*∗∗*^
Cost				
Vaccines are too expensive for me right now	223	1.96 (0.83)	22.4	6
Access				
I do not have time to get a flu vaccination	223	2.22 (0.89)	35.0	4
I do not know where to receive a flu vaccination	222	1.77 (0.81)	19.4	7
Safety				
I believe that as a result of the flu shot I may actually get the flu	223	2.37 (1.00)	47.8	1
I believe that vaccines may have dangerous side effects	221	2.31 (0.96)	41.6	2
Perceived importance				
I was not informed that flu vaccines might be important	222	1.95 (0.82)	26.6	5
I do not believe I am in danger of contracting the flu	222	2.32 (0.85)	39.6	3

^*∗*^Barriers were assessed on a Likert scale of agreement from 1 (Strongly Disagree) to 4 (Strongly Agree) and ranked based on percentages of statement agreement (scores of 3-4).

^*∗∗*^Ranked in order of percent of agreement with the referenced statements.

## References

[1] Sobal J., Loveland F. C. (1982). Infectious disease in a total institution: a study of the influenza epidemic of 1978 on a college campus. *Public Health Reports*.

[2] Uchida M., Tsukahara T., Kaneko M., Washizuka S., Kawa S. (2012). How the H1N1 influenza epidemic spread among university students in Japan: experience from Shinshu University. *American Journal of Infection Control*.

[3] Guh A., Reed C., Gould L. H. (2011). Transmission of 2009 pandemic influenza a (H1N1) at a public university—Delaware, April-May 2009. *Clinical Infectious Diseases*.

[4] Layde P. M., Engelberg A. L., Dobbs H. I. (1980). Outbreak of influenza A/USSR/77 at Marquette University. *Journal of Infectious Diseases*.

[5] Pons V. G., Canter J., Dolin R. (1980). Influenza A/USSR/77 (H1N1) on a university campus. *American Journal of Epidemiology*.

[6] Nichol K. L., D'Heilly S., Ehlinger E. (2005). Colds and influenza-like illnesses in university students: impact on health, academic and work performance, and health care use. *Clinical Infectious Diseases*.

[7] Nichol K. L., D'Heilly S., Ehlinger E. (2006). Burden of upper respiratory illnesses among college and university students: 2002-2003 and 2003-2004 cohorts. *Vaccine*.

[8] Grohskopf L. A., Shay D. K., Shimabukuro T. T. (2013). Prevention and control of seasonal influenza with vaccines: recommendations of the Advisory Committee on Immunization Practices–United States, 2013-2014. *Morbidity and Mortality Weekly Report*.

[9] Couch R. B., Keitel W. A., Cate T. R. (1996). Prevention of influenza virus infections by current inactivated influenza vaccines. *Options for the Control of Influenza III*.

[10] Merrill R. M., Kelley A. T., Cox E., Layman A. B., Layton J. B., Lindsay R. (2010). Factors and barriers influencing influenza vaccination among students at Brigham Young University. *Medical Science Monitor*.

[11] Uddin M., Cherkowski G. C., Liu G., Zhang J., Monto A. S., Aiello A. E. (2010). Demographic and socioeconomic determinants of influenza vaccination disparities among university students. *Journal of Epidemiology and Community Health*.

[12] Poehling K. A., Blocker J., Ip E. H., Peters T. R., Wolfson M. (2012). 2009-2010 seasonal influenza vaccination coverage among college students from 8 universities in North Carolina. *Journal of American College Health*.

[13] Lee S. I., Aung E. M., Chin I. S. (2012). Factors affecting medical students' uptake of the 2009 pandemic influenza A (H1N1) vaccine. *Influenza Research and Treatment*.

[14] http://www.collegeportraits.org/CA/CSUN/characteristics.

[15] Jerant A., Fiscella K., Tancredi D. J., Franks P. (2013). Health insurance is associated with preventive care but not personal health behaviors. *Journal of the American Board of Family Medicine*.

[16] Stone E. G., Morton S. C., Hulscher M. E. (2002). Interventions that increase use of adult immunization and cancer screening services: a meta-analysis. *Annals of Internal Medicine*.

[17] http://www.cdc.gov/vaccines/hcp/vis/vis-statements/flu-largetype.pdf.

[18] Ramsey M. A., Marczinski C. A. (2011). College students' perceptions of H1N1 flu risk and attitudes toward vaccination. *Vaccine*.

[19] Tsuang W. M., Bailar J. C., Englund J. A. (2004). Influenza-like symptoms in the college dormitory environment: a survey taken during the 1999-2000 influenza season. *Journal of Environmental Health*.

[20] Office of Disease Prevention and Health Promotion (2016). *Healthy People 2020*.

